# Event-Triggered Sliding Mode Neural Network Controller Design for Heterogeneous Multi-Agent Systems

**DOI:** 10.3390/s23073477

**Published:** 2023-03-26

**Authors:** Xinhai Shen, Jinfeng Gao, Peter X. Liu

**Affiliations:** 1 School of Information Science and Engineering, Zhejiang Sci-Tech University, Hangzhou 310018, China; 2 Department of Systems and Computer Engineering, Carleton University, Ottawa, ON K1S 5B6, Canada; xpliu@sce.carleton.ca

**Keywords:** multi-agent systems, radial basis function neural network, global sliding mode control, event-triggered mechanism

## Abstract

A class of heterogeneous second-order multi-agent consensus problems is studied, in which an event-triggered method is used to improve the feasibility of the control protocol. The sliding mode control method is used to achieve the robustness of the system. A special type of general radial basis function neural network is applied to estimate the uncertainties. The event-triggered mechanism is introduced to reduce the update frequency of the controller and the communication frequency among the agents. Zeno behavior is avoided by ensuring a lower bound between two adjacent trigger instants. Finally, the simulation results are provided to demonstrate that the time evolution of consensus errors eventually approaches zero. The consensus of multi-agent systems is achieved.

## 1. Introduction

The consensus problem is a fundamental issue in multi-agent systems (MASs) [1]. In recent years, the consensus problems have been widely discussed in many fields, such as the fixed topology [2] and switched topology [3,4]. Moreover, many methods to analyze consensus have been proposed. A class of adaptive fully distributed consensus problems has been investigated for heterogeneous nonlinear MASs [5,6]. The optimization problem of self-balancing robots under depleting battery conditions was analyzed [7]. Additionally, under-actuated systems using the error-magnitude-dependent self-tuning of the cost weighting factor via the adaptive state-space control law has been studied [8].

The sliding mode control scheme (SMCS) is superior to other control schemes in that it switches the modes of controllers with the current state [2,9]. There are various SMC strategies, such as terminal SMC [10,11], dynamic SMC [12] and adaptive SMC [13], and so on. SMC is widely used in practical engineering applications with excellent and robust performance [14], such as stochastic systems [15], robot MASs [16] and heterogeneous nonlinear MASs [17]. Compared with the traditional SMC method, the initial compensation term is utilized in the global sliding mode control scheme (GSMCS) so that the systems with GSMCS can approach the sliding surface at the beginning.

Traditional adaptive control methods require the prior information of the model. By using the self-learning ability of neural networks, the controller does not demand much system information. Thus, neural networks effectively solve the control problem with uncertain models. The practical application then depends on the generalization ability, which determines whether a network is effective or not [18,19,20]. Neural networks have been greatly developed in pattern recognition, signal processing, modeling technology and system control [21,22]. The hidden layer of the neural network adopts the activation function, which can approximate the arbitrary nonlinear function. Among them, radial basis function neural networks (RBFNN) can effectively improve the controller performance when the system has great uncertainty [23]. However, the pressure of parameter estimation increases significantly with an increase in neural network nodes or fuzzy rules. Thus, the minimum parameter learning method is proposed to solve this problem [24]. To address the problem of the low processing power of traditional agricultural machinery design systems in analyzing data, a novel agricultural machinery intelligent design system integrating image processing and knowledge reasoning is constructed [25]. The main advantage of this method is that a single parameter needs to be estimated online, regardless of the number of fuzzy rule bases used. The limitation of minimum parameter learning using radial basis function neural networks is that numerical values are used as parameter estimators. This will cause the estimate to deviate considerably from the true value.

It is noted that continuous communication between follower agents in [26] results in a computing resource waste. Therefore, a method to reduce communication pressure is urgently needed. The event-triggered mechanism (ETM) was proposed to decrease the load of information transmission. There are four types of ETM, which include the event-based sampling mechanism [27], model-based sampling mechanism [28], ETM based on sampled data [29,30] and self-triggered mechanism [31]. There are many applications of ETM, such as the consensus of MASs [32,33,34], sliding mode control [35,36] and convex optimization [37,38]. However, Zeno behavior is a very serious condition in the event triggering mechanism. If Zeno behavior exists in the system, it means that the control of the system is continuous. In this case, the advantages of the event triggering mechanism in saving network resources and reducing the network communication burden cannot be utilized, which means that the design of the event triggering mechanism is unreasonable [39].

Based on the above results, there are few works studying the SMC strategy regarding the minimum parameter learning method under the framework of heterogeneous MASs. This motivates us to design a global sliding mode controller (GSMC) with an event-triggered controller. Compared with some existing results, the event triggering mechanism used in this paper is innovatively combined with global sliding mode control and an RBF neural network. Comparing the exponential event trigger threshold and the constant event trigger threshold, the exponential event trigger threshold can better adapt to the control law and reduce the communication pressure. A strategy based on the minimum parameter learning method is proposed to solve the system uncertainty and the main contributions can be described as follows:

(a) Compared with [40], the single agent system is extended to MASs. In actual production, it is difficult to ensure that every agent has the same dynamic performance. Thus, a class of heterogeneous second-order leader–follower MASs is discussed to make the result more practical. A class of distributed control laws is proposed to enable the follower to approach the leader’s trajectory.
(b) The ETM is inserted into the design of GSMC. The initial compensation term is utilized in GSMC so that the systems with the GSMC scheme can approach the sliding surface at the beginning. Then, the robustness of heterogeneous nonlinear MASs is improved according to their insensitivity to disturbance. The communication pressure between agents is decreased by utilizing ETM.
(c) The online learning ability of RBFNN has been introduced to deal with the uncertainty. Differing from general RBFNN control methods, the proposed control scheme does not need to update all the hidden layer weights. This method reduces the amount of calculation required for each iteration. The time for the system to reach a stable state is decreased.

This article is organized as follows. Firstly, the preliminaries and problem formulation are described in Section 2. In Section 3, a robust controller that is based on the sliding mode mechanism and ETM is presented. Then, the Zeno behavior is excluded. A simulation example is presented in Section 4 and the effectiveness of the proposed method is verified. Finally, some concluding remarks are provided in Section 5.

## 2. Preliminaries and System Statement

### 2.1. Graph Theory

The communication topology of systems is described as G=(V,E). The node set is $V=\left\{v_{1},v_{2},\cdots ,v_{n}\right\}$. E⊂V×V is represented as the edge set. Then, the node *j* is considered as the neighbor of node *i* if node *i* can transmit information to node *j*. The matrix $A=\left[a_{ij}\right]\in R^{N\times N}$ denotes the adjacency matrix. If i=j, then $a_{ij}=0$. The Laplace matrix is defined as $L={\left(l_{ij}\right)}_{N\times N}$. It should be pointed out that $l_{ii}=\sum_{j=1}^{N}a_{ij}$ and the non-diagonal elements are $l_{ij}=-a_{ij}$. The leader–follower system is considered. Meanwhile, the communication topology graph of systems is augmented to $\hat{G}=\left\{\hat{V},\hat{E},\right\}$. $B=diag\left\{b_{1},b_{2},\cdots ,b_{N}\right\}$, which is represented as the input matrix. The Laplace matrix of the follower graph denotes $L_{B}=L+B$. $∥\cdot ∥$ denotes the spectral norm of the matrix. ${∥\cdot ∥}_{F}$ denotes the Frobenius norm of the matrix. ${∥\cdot ∥}_{\infty }$ is the infinite norm of the vector. $\bar{\psi }\left(P\right)$ is the maximal singular value of the matrix *P* and $\underline{\psi }\left(P\right)$ is the minimal singular value of the matrix *P*. $tr\left\{\cdot \right\}$ is the trace of the matrix. $I_{N}$ represents the n-dimensional unit column vector.

There are some necessary lemmas.

**Lemma** **1.** *For ϕ,s∈R and $W,h\in R^{N}$, the inequality $sW^{T}h\leq \frac{1}{2}s^{2}\phi h^{T}h+\frac{1}{2}$ holds.*

**Lemma** **2** ([18])**.** *For sliding mode function $s=c\cdot e+\dot{e}$, a compensating function is designed as $q\left(t\right)$, which satisfies the following conditions:*

*(1)*  *$q\left(0\right)=c\cdot e\left(0\right)+\dot{e}\left(0\right)$;*
*(2)*  *When t→∞, $q\left(t\right)\to \infty$;*
*(3)*  *$q\left(t\right)$ has a bounded first derivative with respect to time,*

*where* $e\left(0\right)$*is the state error. Then,* s→0 *is always established.*

**Lemma** **3** ([40])**.** *For function $H\left(\chi \right)$, $\chi \in \left[0,+\infty \right)$ satisfies the Holder continuous condition. For every $\chi ,\nu \in \left[0,+\infty \right)$, the inequality*

$$H\left(\chi \right)-H\left(\nu \right)\leq \lambda {∥\chi -\nu ∥}_{\infty }$$

*holds and λ is a positive constant.*

**Lemma** **4.**  *If $V:R^{N}\to R$ is a locally positive definite function, $\dot{V}\leq 0$ is obtained in compact set $\Omega_{c}=\left\{x\in R^{N}:V\left(x\right)\leq c\right\}$, where c is a constant. Define $§=\left\{x\in \Omega_{c}:\dot{V}\left(x\right)=0\right\}$. When t→∞, the trajectory approaches the maximum invariant set in *§*. If there is no other solution in *§* except x(t)=0, the origin is asymptotically stable.*

### 2.2. Problem Formulation

Consider a type of leader–follower MAS with *N* agents. The dynamics of agent *i* are denoted as

(1) $$\left\{\begin{array}{cc} {\dot{x}}_{i} & =v_{i}\\ {\dot{v}}_{i} & =f_{i}\left(x\right)+g_{i}\left(x\right)u_{i}+d_{i}\left(x\right) \end{array}\right.$$

where $x={\left[x_{i},v_{i}\right]}^{T}$ represents the state of the system. $u_{i}\in R$ is the control input. $f_{i}\left(\cdot \right)$ and $g_{i}\left(\cdot \right)$ are known continuous smooth functions. $d_{i}\left(\cdot \right)$ is an unknown smooth uncertain function. $g_{i}\left(x\right)\in R^{+}$ is assumed. The dynamic function of the leader is expressed as

(2) $$\left\{\begin{array}{cc} {\dot{x}}_{0} & =v_{0}\\ {\dot{v}}_{0} & =f_{0}\left(x\right)+g_{0}\left(x\right)u_{0} \end{array}\right.$$

The consensus errors are proffered as

(3) $$e_{ix}=\sum_{j=1}^{N}a_{ij}\left(x_{i}-x_{j}\right)+b_{i}\left(x_{i}-x_{0}\right)$$

(4) $$e_{iv}=\sum_{j=1}^{N}a_{ij}\left(v_{i}-v_{j}\right)+b_{i}\left(v_{i}-v_{0}\right)$$

The following definitions are provided: $\varepsilon_{1}={\left[e_{1x},e_{2x},\cdots ,e_{ix}\right]}^{T}$, $\varepsilon_{2}={\left[e_{1v},e_{2v},\cdots ,e_{iv}\right]}^{T}$, $x={\left[x_{1},x_{2},\cdots ,x_{i}\right]}^{T}$, $d={\left[d_{1},d_{2},\cdots ,d_{i}\right]}^{T}$ and then $v={\left[v_{1},v_{2},\cdots ,v_{i}\right]}^{T}$, $\overset{˘}{x}=x-I_{N}⊗x_{0}$, $\overset{˘}{v}=v-I_{N}⊗v_{0}$, $u={\left[u_{1},u_{2},\cdots ,u_{i}\right]}^{T}$, $G=diag\left\{g_{1},g_{2},\cdots ,g_{N}\right\}$ and $B_{L}=L_{B}⊗I_{N}$.

Then, global consensus errors are obtained as

(5) $$\varepsilon_{1}=B_{L}\overset{˘}{x}$$

(6) $$\varepsilon_{2}=B_{L}\overset{˘}{v}$$

According to Equations (5) and (6), the following equations are obtained:

(7) $${\dot{\varepsilon }}_{1}=\varepsilon_{2}$$

(8) $${\dot{\varepsilon }}_{2}=B_{L}\left(F-I_{N}⊗f_{0}+G\cdot u-I_{N}⊗\left(g_{0}\cdot u_{0}\right)+D\right)$$

where $F={\left[f_{1},f_{2},\cdots ,f_{N}\right]}^{T}$ and $D={\left[d_{1},d_{2},\cdots ,d_{N}\right]}^{T}$. According to Lemma 3, the global sliding mode function of *i*-th agent is proposed as

(9) $$\varsigma_{i}\left(t\right)=c_{i}\cdot e_{ix}\left(t\right)+e_{iv}\left(t\right)-\varrho_{i}\left(t\right)$$

where $\varrho_{i}\left(t\right)=\varrho_{i}\left(0\right)e^{-k_{i}t}$. $e_{ix}\left(0\right)$ and $e_{iv}\left(0\right)$ are the initial consensus error. Then, the derivative of *t* can be calculated as

(10) $$\begin{array}{cc} {\dot{\varsigma }}_{i}\left(t\right) & =c_{i}{\dot{e}}_{ix}\left(t\right)+{\dot{e}}_{iv}\left(t\right)-{\dot{\varrho }}_{i}\left(t\right)\\ \; & =f_{i}+g_{i}+d_{i}-{\dot{v}}_{0}+c_{i}{\dot{e}}_{ix}-{\dot{\varrho }}_{i} \end{array}$$

Let $\varsigma \left(t\right)={\left[\varsigma_{1}\left(t\right),\varsigma_{2}\left(t\right),\cdots ,\varsigma_{N}\left(t\right)\right]}^{T}$ and $\dot{\varsigma }\left(t\right)={\left[{\dot{\varsigma }}_{1}\left(t\right),{\dot{\varsigma }}_{2}\left(t\right),\cdots ,{\dot{\varsigma }}_{N}\left(t\right)\right]}^{T}$, and the corresponding sliding mode function and its first-order derivative are designed as

(11) $$\varsigma \left(t\right)=\omega \cdot \varepsilon_{1}\left(t\right)+\varepsilon_{2}\left(t\right)-\varrho \left(t\right)$$

(12) $$\begin{array}{cc} \dot{\varsigma }\left(t\right) & =\omega \cdot {\dot{\varepsilon }}_{1}\left(t\right)+{\dot{\varepsilon }}_{2}\left(t\right)-\dot{\varrho }\left(t\right)\\ \; & =\omega \cdot \varepsilon_{2}+B_{L}\left(F+G\cdot u-I_{N}⊗v_{0}+D\right)-\dot{\varrho }\left(t\right) \end{array}$$

where $\omega =diag\left\{c_{1},c_{2},\cdots ,c_{N}\right\}$. Then, a scheme is proposed that uses neural networks to approximate the uncertainty term of the systems (1). The function of the estimation of $d_{i}\left(x\right)$ is denoted as

(13) $$d_{i}\left(x\right)={W_{i}^{*}}^{T}\cdot h^{i}\left(x\right)+\sigma_{i}\left(x\right)$$

where $W_{i}^{*}={\left[W_{i1}^{*},W_{i2}^{*},\cdots ,W_{im}^{*}\right]}^{T}\in R^{m}$ is the ideal weight vector of RBFNN of agent *i*. *m* is the number of hidden layers. $\sigma_{i}\left(x\right)$ is the estimation error of the RBFNN network.

(14) $$h_{j}^{i}\left(x\right)=\exp\left(-\frac{{∥x-c_{j}^{i}∥}^{2}}{{b_{j}^{i}}^{2}}\right)$$

where *x* is adopted as the input of the neural networks and j=1,2,⋯,m. The center of the corresponding field denotes $c_{j}^{i}$. $b_{j}^{i}$ is the width of the Gaussian function. In addition, $h_{i}\left(x\right)={\left[h_{i1}\left(x\right),h_{i2}\left(x\right),\cdots ,h_{im}\left(x\right)\right]}^{T}$ represents the output Gaussian basis function of agent *i*. The estimated output of the RBFNN is denoted as

(15) $${\hat{\rho }}_{i}\left(x\right)={\hat{W}}_{i}^{*}{}^{T}\cdot h_{i}\left(x\right)$$

where ${\hat{W}}_{i}^{*}$ is the estimated weight. We denote the corresponding radial basis function vector, the error of the RBFNN and the optimal weight matrix as $h\left(x\right)={\left[h_{1}^{T}\left(x\right),\cdots ,h_{N}^{T}\left(x\right)\right]}^{T}$, $W\left(x\right)=blkdiag\left\{W_{1}\left(x\right),\cdots ,W_{N}\left(x\right)\right\}$ and $\sigma \left(x\right)={\left[\sigma_{1}\left(x\right),\cdots ,\sigma_{N}\left(x\right)\right]}^{T}$, and the global output of the RBFNN is written as

(16) $$\rho \left(x\right)=W^{T}\cdot h\left(x\right)+\sigma$$

Meanwhile, the estimation of $\rho \left(x\right)$ is

(17) $$\hat{\rho }\left(x\right)={\hat{W}}^{*}{}^{T}\cdot h\left(x\right)$$

Instead of the weight matrix, an adaptive variable is defined as $\phi_{i}={∥W_{i}∥}_{2}^{2}$ from the minimum parameter learning method. Meanwhile, ${\hat{\phi }}_{i}$ is the estimation of $\phi_{i}$ and ${\tilde{\phi }}_{i}={\hat{\phi }}_{i}-\phi_{i}$. For convenience, we denote $h_{i}^{T}h^{T}$ as $H_{i}$. The adaptive control law of ${\hat{\phi }}_{i}$ is designed as

(18) $${\dot{\hat{\phi }}}_{i}=\frac{\gamma ∥B_{L}∥}{2}\varsigma_{i}^{2}H_{i}-\kappa \gamma {\hat{\phi }}_{i}$$

where γ is any positive constant. Moreover, κ is the positive parameter to be designed.

**Figure 1:**
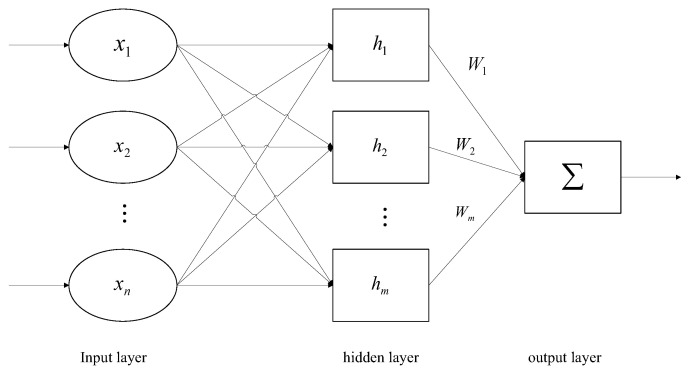
The general structure of the RBF neural network.

**Remark** **1.** *The structure of the RBF neural network is similar to that of a multi-layer forward network. It is generally composed of an input layer, hidden layer and output layer. The first layer is the input layer, which is composed of signal source nodes and transmits signals to the hidden layer. The second layer is the hidden layer, and the transformation function of the hidden layer node is a non-negative nonlinear function with radial symmetry and attenuation to the central point. The third layer is the output layer, which is generally a simple linear function that responds to the input pattern. Figure 1 shows the general structure of the RBF neural network.*

**Remark** **2.** *The width of the Gaussian basis function affects the range of network mapping. It is generally designed to be an appropriate value. According to the input of RBFNN, the center point coordinate vector should keep the Gaussian basis function within the valid mapping range.*

## 3. Main Results

### 3.1. Adaptive Control Law Design with GSMC

In this section, the GSMC using the minimum parameter learning method is designed. The GSMC law forces the target system to follow the ideal sliding mode trajectory. The uncertainties are compensated by using a class of RBFNN methods.

Then, the *i*-th agent of GSMC is proposed as

(19) $$\begin{array}{cc} u_{i}\left(t\right)= & \sum_{i=1,i\neq j}^{N}a_{ij}u_{j}\left(t\right)+\frac{1}{g_{i}}\left[-\varsigma_{i}\hat{\phi }H_{i}/2+v_{0}-c_{i}e_{ix}\right.\\ \; & \left.-f_{i}-\eta_{i}\mathrm{sgn}\left(\varsigma_{i}\right)-\mu_{i}\varsigma_{i}+\varrho_{i}\right] \end{array}$$

where $\eta_{i}$ and $\mu_{i}$ are the positive parameters to be designed and $u_{j}\left(t\right)$ represents the control law of adjacent agent *j*. Let $u\left(t\right)={\left[u_{1}\left(t\right),\cdots ,u_{N}\left(t\right)\right]}^{T}$, $\phi =diag\left\{\phi_{1},\cdots ,\phi_{N}\right\}$, $\hat{\phi }=diag\left\{{\hat{\phi }}_{1},{\hat{\phi }}_{2},\cdots ,{\hat{\phi }}_{N}\right\}$, $\tilde{\phi }=diag\left\{{\tilde{\phi }}_{1},{\tilde{\phi }}_{2},\cdots ,{\tilde{\phi }}_{N}\right\}$, $\mu =diag\left\{\mu_{1},\mu_{2},\cdots ,\mu_{N}\right\}$ and $\eta =diag\left\{\eta_{1},\eta_{2},\cdots ,\eta_{N}\right\}$. The controller $u_{i}\left(t\right)$ presented in this article consists of several parts. The control input of adjacent agents is obtained so that the current agents can gradually reduce the adjoint errors. The RBFNN function can fit the unknown part of the system. Part of the global sliding mode control rate makes the system robust when subjected to external interference. The control protocol of the systems (1)–(2) is developed with

(20) $$\begin{array}{cc} u(t)= & G^{-1}\cdot \left[B_{L}^{-1}\left(-\omega \cdot \varepsilon_{2}-\eta \mathrm{sgn}\left(\varsigma \right)-\mu \varsigma \right)\right.\\ \; & \left.-\frac{1}{2}\varsigma \hat{\phi }h^{T}h-F+v_{0}⊗I_{N}+q\right] \end{array}$$

Substituting (20) into (12), (12) is rewritten as

(21) $$\dot{\varsigma }\left(t\right)=B_{L}\left(W^{T}h+\sigma -\frac{1}{2}\hat{\phi }H\varsigma \right)-\eta \mathrm{sgn}\left(\varsigma \right)-\mu \varsigma$$

**Theorem** **1.** *The leader–follower MAS (1)–(2) is considered and the GSMC (19) with the adaptive minimum parameter learning mechanism (17) is designed. When the time approaches infinity, the consensus of the MASs is achieved.*

**Proof.**  See Appendix A. □

### 3.2. Adaptive Control Law Design with ETM

To minimize the communication pressure of the nonlinear system, the GSMC based on the event triggering mechanism is designed in this section. After the smart sensor receives the current states of the plants, the ETC compares the current system’s states with the last event-triggered system’s states. The state measurement errors between the current moment and event-triggered moment are defined as

(22) $$\Delta_{ix}=x_{i}\left(t_{k}^{i}\right)-x_{i}\left(t\right)$$

(23) $$\Delta_{iv}=v_{i}\left(t_{k}^{i}\right)-v_{i}\left(t\right)$$

Let $t_{k}^{i}$ be the *k*-th triggered instant of *i*-th agent. The measurement errors of the leader are expressed as

(24) $$\Delta_{0x}=x_{0}\left(t_{k}^{0}\right)-x_{0}\left(t\right)$$

(25) $$\Delta_{0v}=v_{0}\left(t_{k}^{0}\right)-v_{0}\left(t\right)$$

where $t_{k}^{0}$ is the moment when the ETC of the leader works *k* times. The state error between the last triggering instant and the current instant is denoted as $\Delta_{i}={\left[\Delta_{ix},\Delta_{iv}\right]}^{T}$. According to Theorem 1, the control strategy is rewritten as

(26) $$\left\{\begin{array}{cc} u_{i}\left(t\right) & =u_{i}\left(t_{k}^{i}\right)\\ t_{k+1}^{i} & =\inf\left\{t>t_{k}^{i}|∥\Delta_{i}∥\leq e^{-\alpha t}\vee t-t_{k}^{i}>T\right\} \end{array}\right.$$

where α is a designed positive constant. $u_{i}\left(t\right)$ represents the output of the agent *i* controller and $u_{i}\left(t_{k}^{i}\right)$ is equivalent to control at time $t_{k}^{i}$. $t_{k+1}^{i}$ indicates the next triggered instant. In addition, there is upper bound time *T* between the instant of event-triggered $t_{k}^{i}$ and execution time *t*. Moreover, the controller does not update until the measurement error satisfies $∥\Delta_{i}∥\leq e^{-\alpha t}$. Define $\Delta ={\left[{∥\Delta_{1}∥}_{\infty },{∥\Delta_{2}∥}_{\infty },\cdots ,{∥\Delta_{N}∥}_{\infty }\right]}^{T}$. According to the corresponding control strategy, the function of the control of *i*-th agent is rewritten as

(27) $$\begin{array}{cc} u_{i}\left(t\right)= & \sum_{i=1}^{N}a_{ij}u_{j}\left(t_{k}^{i}\right)+\frac{1}{g_{i}\left(x\left(t_{k}^{i}\right)\right)}\\ \; & \left(-\varsigma_{i}\left(t_{k}^{i}\right)\hat{\phi }\left(t_{k}^{i}\right)h_{i}^{T}\left(x_{i}\left(t_{k}^{i}\right)\right)h_{i}\left(x_{i}\left(t_{k}^{i}\right)\right)/2+v_{0}\left(t_{k}^{i}\right)\right.\\ \; & \left.-c_{i}e_{ix}\left(t_{k}^{i}\right)-f_{i}\left(x\left(t_{k}^{i}\right)\right)-\mu_{i}\varsigma_{i}\left(t_{k}^{i}\right)+\varrho_{i}\left(t_{k}^{i}\right)\right) \end{array}$$

where $\varsigma_{i}\left(t_{k}^{i}\right)=c_{i}\cdot e_{ix}\left(t_{k}^{i}\right)+e_{iv}\left(t_{k}^{i}\right)-\varrho_{i}\left(t_{k}^{i}\right)$ is *i*-th agent in the sliding mode function. Meanwhile,  $v_{0}\left(t_{k}^{i}\right)=f_{0}\left(t_{k}^{i}\right)+g_{0}\left(t_{k}^{i}\right)u_{0}\left(t_{k}^{i}\right)$ is the leader velocity function.

The global function of the controller is calculated as

(28) $$\begin{array}{cc} u(t)= & G^{-1}\left(x\left(t_{k}\right)\right)\cdot \left[B_{L}^{-1}\left(-\omega \cdot \varepsilon_{2}\left(t_{k}\right)-\eta \mathrm{sgn}\left(\varsigma \left(t_{k}\right)\right)-\mu \varsigma \left(t_{k}\right)\right)\right.\\ \; & \left.-\frac{1}{2}\varsigma \left(t_{k}\right)\hat{\phi }\left(t_{k}\right)h^{T}h-F\left(x\left(t_{k}\right)\right)+v_{0}\left(t_{k}\right)+\varrho \left(t_{k}\right)\right] \end{array}$$

where

$\varepsilon_{2}\left(t_{k}\right)={\left[e_{1v}\left(t_{k}^{1}\right),e_{2v}\left(t_{k}^{2}\right),\cdots ,e_{Nv}\left(t_{k}^{N}\right)\right]}^{T}$ is the corresponding error of the system velocity;
$F\left(x_{k}\right)={\left[F\left(x_{k}^{1}\right)F\left(x_{k}^{2}\right),\cdots ,F\left(x_{k}^{N}\right)\right]}^{T}$ is the dynamic function term of the system;
$G\left(x_{k}\right)=diag\left\{g_{1}\left(x_{k}^{1}\right),g_{2}\left(x_{k}^{2}\right),\cdots ,g_{N}\left(x_{k}^{N}\right)\right\}$ is the dynamic gain of the system;
$v_{0}\left(t_{k}\right)={\left[v_{0}\left(t_{k}^{1}\right),v_{0}\left(t_{k}^{2}\right),\cdots ,v_{0}\left(t_{k}^{N}\right)\right]}^{T}$ is the velocity output of the leader in various event-triggered times;
$\varsigma \left(t_{k}\right)={\left[\varsigma_{1}\left(t_{k}^{1}\right),\varsigma_{2}\left(t_{k}^{2}\right),\cdots ,\varsigma_{N}\left(t_{k}^{N}\right)\right]}^{T}$ is the sliding mode function of the system;
$sgn\left(\varsigma \left(t_{k}\right)\right)={\left[sgn\left(\varsigma_{1}\left(t_{k}^{1}\right)\right),sgn\left(t_{k}^{2}\right),\cdots ,sgn\left(\varsigma_{1}\left(t_{k}^{N}\right)\right)\right]}^{T}$ is the sign function of the sliding mode function;
$H\left(x\left(t_{k}\right)\right)={\left[H_{1}\left(x\left(t_{k}^{1}\right)\right),H_{2}\left(x\left(t_{k}^{2}\right)\right),\cdots ,H_{N}\left(x\left(t_{k}^{N}\right)\right)\right]}^{T}$ is the output Gaussian basis function of the system;
$\varrho \left(t_{k}\right)={\left[\varrho_{1}\left(t_{k}^{1}\right),\varrho_{2}\left(t_{k}^{2}\right),\cdots ,\varrho_{N}\left(t_{k}^{N}\right)\right]}^{T}$ is the uncertainty term of the system;
$\omega =diag\left\{c_{1},c_{2},\cdots ,c_{N}\right\}$ and $\mu =diag\left\{\mu_{1},\mu_{2},\cdots ,\mu_{N}\right\}$ are the control parameters.

**Theorem** **2.** *Considering the leader–follower MASs (1)–(2), the sliding mode control strategy (19) is proposed with the adaptive minimum parameter learning mechanism (17) and event-triggered law (32). When the time approaches infinity, the consensus of the systems is achieved. Finally, the sliding mode states of agent i converge to the ultimate bound*

(29) $$\Omega =\left\{x_{i}\left(t\right)\in R,v_{i}\left(t\right)\in R:\left|\varsigma_{i}\left(t\right)\right|\leq \vartheta \right\}$$

**Proof.**  See Appendix B. □

**Theorem** **3.** *Considering the nonlinear systems (1)–(2) with the GSMC (34) and the event-triggered strategy (32), the interval between two triggered instants is $T_{k}^{i}=t_{k+1}^{i}-t_{k}^{i}$. Meanwhile, the positive lower bound is given as*

(30) $$T_{k}^{i}\geq \frac{1}{\bar{\lambda_{0}}}In\left(1+\frac{{\bar{\lambda }}_{0}}{\bar{n}}\right)$$

**Proof.**  See Appendix C. □

## 4. Simulations

A simulation of a flexible-joint manipulator system that contains a leader and four followers is presented.

A type of heterogeneous second-order flexible-joint manipulator system is considered, and the control goal is to enable the manipulator with different sizes to follow the same desired trajectory. The physical model is borrowed from [40], where

(31) $$I_{i}{\ddot{q}}_{i}+K_{i}{\dot{q}}_{i}+m_{i}gl_{i}=u_{i\tau }+d_{i\tau }$$

where i=1,2,3,4. $q_{i}\in R$ represents the horizontal angular position of the connecting rod of the agent *i*. ${\dot{q}}_{i}$, ${\ddot{q}}_{i}$ are the angle velocity and acceleration of the agent *i*. The moment of inertia is proposed as $I_{i}=4m_{i}l_{i}^{2}/3$. In addition, $m_{i}$ is the *i*-th agent manipulator mass, and $l_{i}$ is the distance from the center of mass of the manipulator linked to the center of the connecting rod. *g* and $u_{i\tau }$ are the acceleration of gravity and the control torque input, respectively. $K_{i}$ is the viscous friction coefficient of the agent *i*. $d_{i\tau }$ denotes the uncertain external disturbances and the unknown system parameter. In order to reduce the impact of external distractions, the virtual desired leader is considered as

(32) $$I_{0}{\ddot{q}}_{0}+K_{0}{\dot{q}}_{0}+m_{0}gl_{0}=u_{0\tau }$$

where $I_{0}=4m_{0}l_{0}^{2}/3$ and $K_{0}$ are the inertial moment and viscous friction coefficient of the leader. The mass of the leader is denoted as $m_{0}$. $l_{0}$ is the distance from the center of mass of the manipulator linked to the center of the connecting rod for the leader. The ideal control torque input is designed as $u_{0\tau }=cos\left(0.1t\right)$ to force the follower to approach the desired trajectory. The control goal is to enable the followers to approach the position of the leader as quickly as possible.

Denoting $x_{i}\left(t\right)=q_{i},v_{i}\left(t\right)={\dot{q}}_{i}$, the dynamic function of the proposed leader–follower MASs is written as

(33) $$\left\{\begin{array}{cc} {\dot{x}}_{i} & =v_{i}\\ {\dot{v}}_{i} & =f_{i}\left(x_{i},v_{i}\right)+g_{i}\left(x_{i},v_{i}\right)u_{i}+d_{i}\left(x_{i},v_{i}\right) \end{array}\right.$$

where $f_{i}\left(x_{i},v_{i}\right)=-3I_{i}x_{i}/\left(4m_{i}l_{i}^{2}\right)-3g/\left(4l_{i}\right)$ and $g_{i}\left(x_{i},v_{i}\right)\;=3/\left(4m_{i}l_{i}^{2}\right)$, i=0,1,2,3,4. Then, $d_{i}\left(x_{i},v_{i}\right)=-3d_{i\tau }/\left(4m_{i}l_{i}^{2}\right)$, i=1,2,3,4.

**Figure 2:**
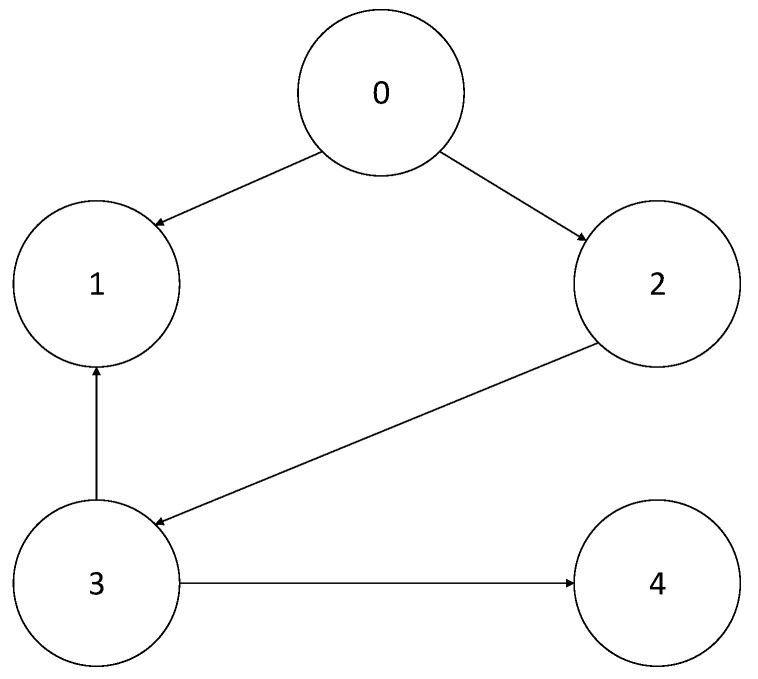
Graph of the communication topology.

In the communication topological graph in Figure 2, the leader node is zero, and the follower nodes are denoted as node one to node four. The Laplacian matrix is written as

$$\begin{array}{c} W=\left[\begin{array}{cccc} 1 & 0 & -1 & 0\\ 0 & 0 & 0 & 0\\ 0 & -1 & 1 & 0\\ 0 & 0 & -1 & 1 \end{array}\right] \end{array}$$

$B=diag\left\{b_{1},b_{2},b_{3},b_{4}\right\}=\left\{1,1,0,0\right\}$ is the input matrix. The dynamic parameters are given as $m_{0}=m_{1}=m_{2}=m_{3}=m_{4}=1$ kg; $l_{0}=l_{1}=0.25$ m, $l_{2}=0.3$ m, $l_{3}=0.35$ m, $l_{4}=0.4$ m; $K_{0}=K_{1}=K_{2}=K_{3}=K_{4}=2$; g=9.8 m/s${}^{2}$. The angular position and velocity at the initial time are denoted as ${\left[x_{0}\left(0\right),v_{0}\left(0\right)\right]}^{T}={\left[0,0\right]}^{T}$, ${\left[x_{1}\left(0\right),v_{1}\left(0\right)\right]}^{T}={\left[0.5,0.5\right]}^{T}$, ${\left[x_{2}\left(0\right),v_{2}\left(0\right)\right]}^{T}={\left[1,1\right]}^{T}$, ${\left[x_{3}\left(0\right),v_{3}\left(0\right)\right]}^{T}={\left[1.5,1.5\right]}^{T}$, ${\left[x_{4}\left(0\right),v_{4}\left(0\right)\right]}^{T}={\left[2,2\right]}^{T}$. The number of hidden layers is chosen as 5. Then, the parameters of RBFNN are given as $c_{j}^{1}=c_{j}^{2}=c_{j}^{3}=c_{j}^{4}$ in which $c_{1}^{i}={\left[-2,-2\right]}^{T}$, $c_{2}^{i}={\left[-1,-1\right]}^{T}$, $c_{3}^{i}={\left[0,0\right]}^{T}$, $c_{4}^{i}={\left[1,1\right]}^{T}$, $c_{5}^{i}={\left[2,2\right]}^{T}$ and $b_{i}^{j}=1$.

The sliding surface is defined as

(34) $${\dot{\varsigma }}_{i}=f_{i}+g_{i}u_{i}+d_{i}-{\dot{v}}_{0}+c_{i}\cdot e_{ix}-{\dot{\varrho }}_{i}$$

**Figure 3:**
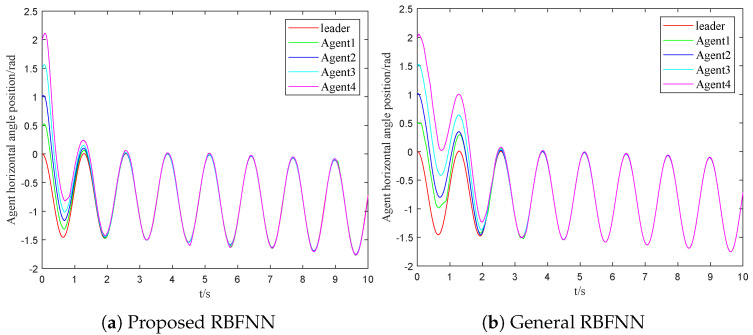
The comparison of the response curves for the horizontal angular position.

**Figure 4:**
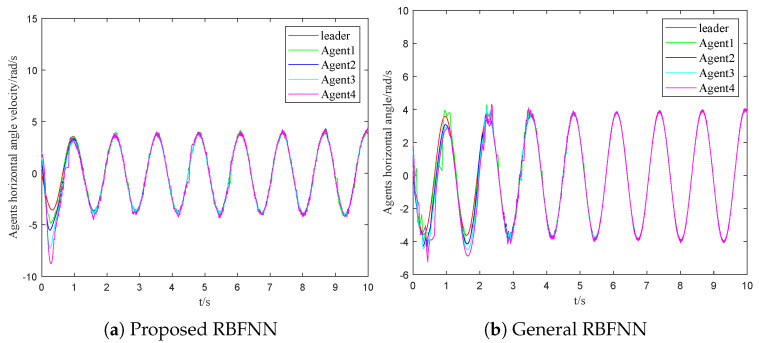
The comparison of response curves for horizontal angular velocity.

Relying on Theorem 2, the continuous control law is proposed as

(35) $$\begin{array}{cc} u_{i\tau }\left(t\right)= & \sum_{i=1}^{N}a_{ij}u_{j\tau }+\left(-\varsigma_{i}\left(t_{k}^{i}\right){\hat{\phi }}_{i}\left(t_{k}^{i}\right)h_{i}^{T}\left(x_{i}\left(t_{k}^{i}\right)\right)\right.\\ & h_{i}\left(x_{i}\left(t_{k}^{i}\right)\right)/2+v_{0}\left(t_{k}^{i}\right)-c_{i}e_{ix}\left(t_{k}^{i}\right)\\ \; & \left.-f_{i}\left(x\left(t_{k}^{i}\right)\right)-\mu_{i}\varsigma_{i}\left(t_{k}^{i}\right)+\varrho_{i}\left(t_{k}^{i}\right)\right)/g_{i}\left(x\left(t_{k}^{i}\right)\right) \end{array}$$

where $u_{j\tau }$ is the control law of the adjacent agent *j*. The assumed external disturbance and uncertainty can be denoted as $d_{i\tau }=v_{i}sin\left(x_{i}\right)+0.1cos\left(t\right)$. Figure 3a and Figure 4a are the response curves of the horizontal angular position and the angular velocity, respectively. From these figures, the asymptotic consensus is achieved.

From Table 1, the whole sampling time is 10,000 and the execution times of the agents are 4839, 3890, 4366, 4815, respectively. As a result, the proposed method reduces the stress of communication.

The results obtained by different event triggering thresholds are shown in Table 2. From the table, the larger the event trigger threshold, the more times the event is triggered but the shorter the stabilization time. Considering the stability of operation and the minimum communication pressure, the event triggering threshold should be selected to be around 1. The threshold selected in this simulation is 0.8. The execution effects of the other agents are similar. In order to display the results as easily as possible, only the running result of agent 1 is listed in the table. The SMC with the general RBFNN is described as

(36) $$\begin{array}{cc} {\bar{u}}_{ir}\left(t\right)= & \sum_{i=1}^{N}a_{ij}{\bar{u}}_{jr}\left(t_{k}^{i}\right)+\left(-\varsigma_{i}\left(t_{k}^{i}\right){\hat{W}}^{T}\left(t_{k}^{i}\right)h_{i}\left(x_{i}\left(t_{k}^{i}\right)\right)/2\right.\\ \; & +v_{0}\left(t_{k}^{i}\right)-c_{i}e_{ix}\left(t_{k}^{i}\right)-f_{i}\left(x\left(t_{k}^{i}\right)\right)-\mu_{i}\varsigma_{i}\left(t_{k}^{i}\right)\\ \; & \left.+\varrho_{i}\left(t_{k}^{i}\right)\right)/g_{i}\left(x\left(t_{k}^{i}\right)\right) \end{array}$$

**Figure 5:**
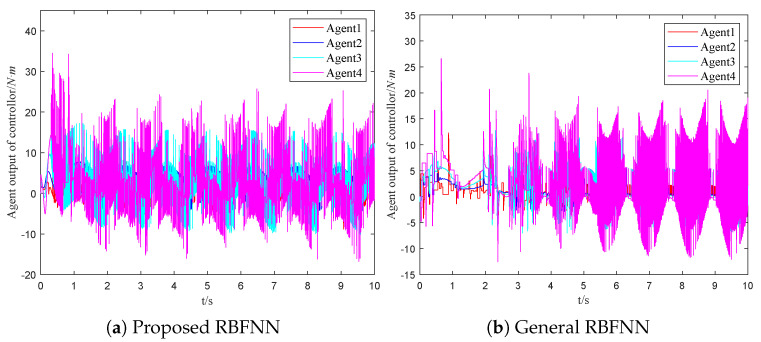
The comparison of the controllers.

The time evolutions of the horizontal angular position and the angular velocity are shown in Figure 3b and Figure 4b, respectively. Then, the time evolutions of the control output are shown in Figure 5b. Comparing Figure 3a with Figure 3b, the followers take around four seconds to approach the trajectory of the leader with the SMC based on the general RBFNN. On the other hand, two seconds will be required by followers with the proposed scheme to achieve a similar effect. Comparing Figure 4a and Figure 4b, the velocity change of the scheme mentioned flattens out after two seconds. In the general RBFNN scheme, the velocity changes more dramatically at the same time. As a result, the dynamic performance of the systems under different control schemes is clearly demonstrated.

The event triggering mechanism used in this paper is innovatively combined with global sliding mode control and radial basis function neural networks. Moreover, the exponential event trigger threshold can better adapt to the control law and reduce the communication pressure. A strategy based on the minimum parameter learning method is proposed to solve the system uncertainty. According to Figure 3, Figure 4 and Figure 5, the dynamic performance of the systems under different control schemes is clearly demonstrated.

**Figure 6:**
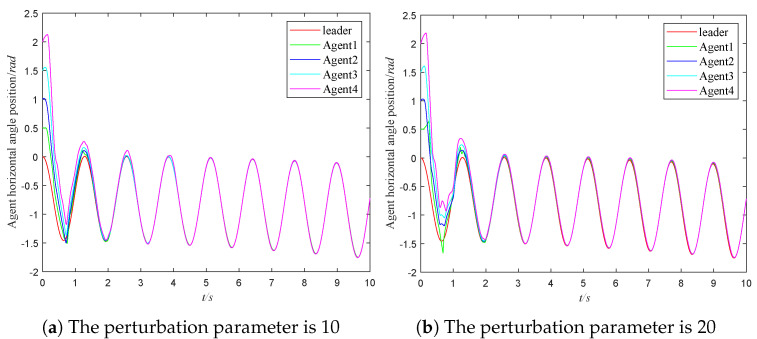
The performance of the system under different non-vanishing disturbances.

In order to verify the control performance of the proposed controller under a non-vanishing disturbance, a bounded non-vanishing disturbance is given in the simulation. The status image of the system is given in Figure 6. To compare the performance of the system under different non-vanishing disturbances, the two types of disturbances are set as 10 and 20, respectively. It can be seen from Figure 6 that when the disturbance increases, the fluctuation of the system increases in the initial operating state. The MASs will eventually reach a consensus.

This section describes numerical simulations to demonstrate the effectiveness of the proposed method. Therefore, the time evolutions of consensus errors eventually approach zero. The consensus of MASs (1)–(2) can be achieved. The results indicate that the designed control protocol is efficient in reducing the adjustment time and overshoot.

## 5. Conclusions

The consensus for heterogeneous second-order MASs is analyzed. Firstly, the SMC based the ON RBFNN scheme is designed. Then, the robustness of the method is analyzed. The RBFNN is used to approximate and compensate for the uncertainty of the system. Secondly, ETM is used so that the transmission time is reduced significantly. In addition, the Zeno behavior is eliminated by a control law. Finally, the simulation results show the superiority of the given method. Moreover, a comparison between the general RBFNN method and the proposed strategy is performed. However, the sliding mode controller still has shortcomings. Due to the discontinuous switching characteristic of the GSMC scheme, chattering occurs in the system. In addition, the agent consensus problem under network attacks is studied based on the results of [41,42].

## Figures and Tables

**Table 1:** Communication decrease for each agent.

Controller	Execution Time	Communication Decrease
Time Mechanism	10,000	-
agent 1	4839	51.6%
agent 2	3890	61.1%
agent 3	4366	56.3%
agent 4	4815	51.8%

**Table 2:** Times of execution under different event triggering thresholds.

ET Thresholds	Execution Time	Communication Decrease	Stabilization Time
1	6686	33.14%	3.2
0.5	3598	64.02%	5.9
5	9323	6.77%	1.5

## Data Availability

https://github.com/shenxinhai/Thesis-code.git.

## Abbreviations

**Abbreviation**	**Complete Phrase**
MASs	multi-agent systems
SMCS	sliding mode control scheme
SMC	sliding mode controller
GSMCS	global sliding mode control scheme
GSMC	global sliding mode controller
ETM	event-triggered mechanism
RBFNN	radial basis function neural network

## Appendix A. Proof of Theorem 1

**Proof.** We choose the Lyapunov function $L=L_{1}+L_{2}$, where $L_{1}=\frac{1}{2}\varsigma^{T}\varsigma$ and $L_{2}=\frac{1}{2\gamma }tr\left\{{\tilde{\phi }}^{T}\tilde{\phi }\right\}$. $L_{1}$ represents the robustness of the sliding mode term and $L_{2}$ represents the robustness of the minimum parameter learning method. In the above equation, γ>0. Then, taking the time derivative of $L_{1}$ as

(A1) $$\begin{array}{cc} {\dot{L}}_{1}= & \varsigma^{T}\dot{\varsigma }\\ = & \varsigma^{T}\left(B_{L}\left(W^{T}h+\sigma -\frac{1}{2}\hat{\phi }H\varsigma \right)-\eta \mathrm{sgn}\left(\varsigma \right)-\mu \varsigma \right)\\ \leq & ∥B_{L}∥\left(∥\sigma ∥∥\varsigma ∥+\frac{1}{2}N-\frac{1}{2}tr\left\{{\tilde{\phi }}^{T}\varsigma \varsigma^{T}H\right\}\right)-∥\eta ∥∥\varsigma ∥-∥\mu ∥{∥\varsigma ∥}^{2} \end{array}$$

According to the definition of RBFNN parameter $\phi_{i}$, it is obvious that the time derivative of $\phi_{i}$ is zero. Thus, the second term of the Lyapunov–Krasovskii function is taken as a derivative as

(A2) $$\begin{array}{cc} {\dot{L}}_{2}= & \frac{1}{\gamma }\mathrm{tr}\left\{{\tilde{\phi }}^{T}\dot{\hat{\phi }}\right\}\\ = & \frac{1}{\gamma }\mathrm{tr}\left\{{\tilde{\phi }}^{T}\left(\frac{\gamma }{2}∥B_{L}∥\varsigma \varsigma^{T}H-\kappa \gamma \hat{\phi }\right)\right\}\\ = & \frac{1}{2}∥B_{L}∥tr\left\{{\tilde{\phi }}^{T}\varsigma \varsigma^{T}H\right\}-\kappa tr\left\{{\tilde{\phi }}^{T}\hat{\phi }\right\}\\ \leq & \frac{1}{2}∥B_{L}∥tr\left\{{\tilde{\phi }}^{T}\varsigma \varsigma^{T}H\right\}-\kappa ∥\hat{\phi }{∥}_{F}{∥\tilde{\phi }∥}_{F} \end{array}$$

Substituting (A1), (A2) and (17) into the first derivative of the Lyapunov–Krasovskii function, the following inequality is acquired:

(A3) $$\dot{L}\leq -∥\mu ∥{∥\varsigma ∥}^{2}-\kappa {∥\hat{\phi }∥}_{F}{∥\tilde{\phi }∥}_{F}+\frac{N}{2}∥B_{L}∥$$

Choosing $\kappa =\frac{2∥\mu ∥}{\gamma }$, it follows that

(A4) $$\begin{array}{cc} \dot{L} & \leq -∥\mu ∥{∥\varsigma ∥}^{2}-\frac{∥\mu ∥}{\gamma }\left({∥\tilde{\phi }∥}_{F}^{2}-{∥\phi ∥}_{F}^{2}\right)+\frac{N}{2}∥B_{L}∥\\ \; & =-2∥\mu ∥\left(\frac{1}{2\gamma }∥\tilde{\phi }{∥}_{F}^{2}+\frac{1}{2}{∥\varsigma ∥}^{2}\right)+\frac{N}{2}∥B_{L}∥+\frac{\kappa }{2}{∥\phi ∥}_{F}^{2}\\ \; & =-2∥\mu ∥\left(\frac{1}{2}\varsigma^{T}\varsigma +\frac{1}{2\gamma }\mathrm{tr}\left\{{\tilde{\phi }}^{T}\tilde{\phi }\right\}\right)+\frac{N}{2}∥B_{L}∥+\frac{\kappa }{2}{∥\phi ∥}_{F}^{2}\\ \; & =-2∥\mu ∥L+Q \end{array}$$

where $Q=\frac{N}{2}∥B_{L}∥+\frac{\kappa }{2}{∥\phi ∥}_{F}^{2}$. *L* is bounded and the differential inequality function can be solved as

(A5) $$L\leq \frac{Q}{2R}+\left(L\left(0\right)-\frac{Q}{2R}\right)e^{-2Rt}$$

where $R=∥\mu ∥$. Therefore, ${∥\tilde{\phi }∥}_{F}$ is bounded. Because $\phi_{i}$ is a constant, ${∥\hat{\phi }∥}_{F}$ is bounded. From the inequality (A5), the states can converge to a bounded value as the time tends to infinity and other signals are ultimately bounded according to the invariance principle.

(A6) $$\lim_{t\to \infty }L=\frac{Q}{2R}=\frac{\frac{\kappa }{2}{∥\phi ∥}_{F}^{2}+\frac{1}{2}N∥B_{L}∥}{2R}=\frac{{∥\phi ∥}_{F}^{2}}{2\gamma }+\frac{N∥B_{L}∥}{4R}$$

Thus, it is concluded that the trajectories of the system states are asymptotically stable and all signals are ensured to be bounded. In conclusion, the follower agent systems (1) converge to the leader system state (2) as the time approaches infinity. □

## Appendix B. Proof of Theorem 2

**Proof.** We choose $L_{3}=L_{4}+L_{5}$ as the Lyapunov–Krasovskii function, where $L_{4}=\frac{1}{2}\varsigma^{T}\varsigma$ and $L_{5}=\frac{1}{2\gamma }tr\left\{{\tilde{\phi }}^{T}\tilde{\phi }\right\}$. $L_{4}$ represents the robustness of the sliding mode term, and $L_{5}$ represents the robustness of the minimum parameter learning method. Then, we take the derivative of $L_{4}$ as follows:

(A7) $$\begin{array}{cc} {\dot{L}}_{4}= & \varsigma^{T}\left(t\right)\dot{\varsigma }\left(t\right)\\ = & \varsigma^{T}\left(B_{L}\left(W^{T}h+\sigma \right)+\omega \cdot \varepsilon_{2}\left(t\right)-\varrho \left(t\right)\right.\\ \; & \left.+B_{L}\left(F\left(x\right)+G\left(x\right)\cdot u\left(t\right)-V_{0}\left(t\right)\right)\right)\\ \leq & \frac{1}{2}\varsigma^{T}B_{L}\phi H\varsigma +\frac{N}{2}∥B_{L}∥-\frac{1}{2}\varsigma^{T}B_{L}\sigma -\eta \mathrm{sgn}\left(\varsigma \right)-\mu \varsigma \\ \leq & ∥B_{L}∥\left(\left(∥\sigma ∥+\frac{1}{2}N\right)∥\varsigma ∥+\frac{1}{2}{∥H∥}_{F}∥\tilde{\phi }{∥}_{F}{∥\varsigma ∥}^{2}\right)\\ \; & -∥\eta ∥∥\varsigma ∥-{∥\mu ∥∥\varsigma ∥}^{2} \end{array}$$

Let ${\hat{g}}_{i}\left(x\right)=g_{i}^{-1}\left(x\right)$. According to the assumption and property of $g\left(x\right),f\left(x\right)$ and $h\left(x\right)$, there are positive constants $\lambda_{0i},\lambda_{1i},\lambda_{2i},\lambda_{3i}$ that satisfy the following inequality:

(A8) $${\hat{g}}_{i}\left(x\right)-{\hat{g}}_{i}\left(x\left(t_{k}^{i}\right)\right)\leq \lambda_{0i}{∥x-x\left(t_{k}^{i}\right)∥}_{\infty }$$

(A9) $$f_{i}\left(x\right)-f_{i}\left(x\left(t_{k}^{i}\right)\right)\leq \lambda_{1i}{∥x-x\left(t_{k}^{i}\right)∥}_{\infty }$$

(A10) $$g_{i}\left(x\right)-g_{i}\left(x\left(t_{k}^{i}\right)\right)\leq \lambda_{2i}{∥x-x\left(t_{k}^{i}\right)∥}_{\infty }$$

(A11) $$H_{i}\left(x\right)-H_{i}\left(x\left(t_{k}^{i}\right)\right)\leq \lambda_{3i}{∥x-x\left(t_{k}^{i}\right)∥}_{\infty }$$

For a clear analysis, the first time derivative of $L_{4}$ is divided into several parts. Firstly,

(A12) $$\begin{array}{cc} \; & \varsigma^{T}B_{L}F\left(x\right)-\varsigma^{T}B_{L}G\left(x\right)G^{-1}\left(x_{k}\right)B_{L}^{-1}F\left(x_{k}\right)\\ = & \varsigma^{T}B_{L}F\left(x\right)-\varsigma^{T}B_{L}F\left(x_{k}\right)+\varsigma^{T}B_{L}F\left(x_{k}\right)-\varsigma^{T}B_{L}G\left(x\right)G^{-1}\left(x_{k}\right)F\left(x_{k}\right)\\ \leq & N∥B_{L}∥∥\lambda_{1}∥{∥\Delta ∥}_{\infty }∥\varsigma ∥+\bar{\psi }\left(B_{L}\right)/\underline{\psi }\left(B_{L}\right)N\lambda_{0}∥\Delta ∥{∥F\left(x_{k}\right)∥}_{F}∥\varsigma ∥\\ = & \zeta_{1}∥\varsigma ∥ \end{array}$$

where $\zeta_{1}=N∥B_{L}∥∥\lambda_{1}∥{∥\Delta ∥}_{\infty }+\bar{\psi }\left(B_{L}\right)/\underline{\psi }\left(B_{L}\right)N\lambda_{0}∥\Delta ∥{∥F\left(x_{k}\right)∥}_{F}$. By using a similar method, (A6) and (A7) are obtained as follows:

(A13) $$\begin{array}{cc} \; & \varsigma^{T}\omega \cdot \varepsilon_{2}\left(t\right)-\varsigma^{T}B_{L}G\left(x\right)G^{-1}\left(x_{k}\right)B_{L}^{-1}\omega \cdot \varepsilon_{2}\left(t_{k}\right)\\ = & \varsigma^{T}\omega \cdot \varepsilon_{2}\left(t\right)-\varsigma^{T}\omega \cdot \varepsilon_{2}\left(t_{k}\right)+\varsigma^{T}\omega \cdot \varepsilon_{2}\left(t_{k}\right)\\ \; & -\varsigma^{T}B_{L}G\left(x\right)G^{-1}\left(x_{k}\right)B_{L}^{-1}\omega \cdot \varepsilon_{2}\left(t_{k}\right)\\ \leq & N∥B_{L}∥∥\omega ∥\cdot \left({∥\Delta ∥}_{\infty }+{∥\Delta_{0}∥}_{\infty }\right)∥\varsigma ∥\\ \; & +\bar{\varphi }\left(B_{L}\right)/\underline{\varphi }\left(B_{L}\right)∥\omega ∥{∥G(x)∥}_{F}∥\lambda_{0}∥{∥\Delta ∥}_{\infty }\\ \; & {∥F\left(x_{k}\right)∥}_{F}{∥\varepsilon_{2}\left(t_{k}\right)∥}_{F}∥\varsigma ∥=\zeta_{2}∥\varsigma ∥ \end{array}$$

where $\zeta_{2}=N∥B_{L}∥∥\omega ∥\cdot \left({∥\Delta ∥}_{\infty }+{∥\Delta_{0}∥}_{\infty }\right)∥\varsigma ∥+\bar{\varphi }\left(B_{L}\right)/\underline{\varphi }\left(B_{L}\right)∥\omega ∥{∥G(x)∥}_{F}\cdot ∥\lambda_{0}∥∥\Delta ∥{∥F\left(x_{k}\right)∥}_{F}{∥\varepsilon_{2}\left(x_{k}\right)∥}_{F}$.

(A14) $$\begin{array}{cc} \; & \varsigma^{T}B_{L}G\left(x\right)G^{-1}\left(x_{k}\right)B_{L}^{-1}\varrho \left(t_{k}\right)-\varsigma^{T}\varrho \left(t\right)\\ = & \varsigma^{T}B_{L}G\left(x\right)G^{-1}\left(x_{k}\right)B_{L}^{-1}\varrho \left(t_{k}\right)\\ \; & -\varsigma^{T}\varrho \left(t_{k}\right)+\varsigma^{T}\varrho \left(t_{k}\right)-\varsigma^{T}\varrho \left(t\right)\\ \leq & \bar{\varphi }\left(B_{L}\right)/\underline{\varphi }\left(B_{L}\right){∥G\left(x\right)∥}_{F}∥\lambda_{0}∥{∥\Delta ∥}_{\infty }∥\varsigma ∥\\ = & \zeta_{3}∥\varsigma ∥ \end{array}$$

where $\zeta_{3}=\bar{\varphi }\left(B_{L}\right)\underline{\varphi }\left(B_{L}\right){∥G\left(x\right)∥}_{F}∥\lambda_{0}∥{∥\Delta ∥}_{\infty }$. From the function of $\varepsilon_{2}\left(t\right)$, $\varepsilon_{2}\left(t\right)=B_{L}\tilde{v}$

(A15) $$\begin{array}{cc} \; & \varsigma^{T}B_{L}V_{0}\left(t\right)-\varsigma^{T}B_{L}G\left(x\right)G^{-1}\left(x_{k}\right)B_{L}^{-1}V_{0}\left(t_{k}\right)\\ = & \varsigma^{T}B_{L}V_{0}\left(t\right)-\varsigma^{T}B_{L}V_{0}\left(t_{k}\right)+\varsigma^{T}B_{L}V_{0}\left(t_{k}\right)\\ \; & -\varsigma^{T}B_{L}G\left(x\right)G^{-1}\left(x_{k}\right)B_{L}^{-1}V_{0}\left(t_{k}\right)\\ \leq & N∥B_{L}∥{∥\Delta ∥}_{\infty }∥\varsigma ∥+\bar{\varphi }\left(B_{L}\right)/\underline{\varphi }\left(B_{L}\right)∥{∥G(x)∥}_{F}\\ \; & ∥\lambda_{0}∥{∥\Delta ∥}_{\infty }∥V_{0}\left(t_{k}\right)∥∥\varsigma ∥=\zeta_{4}∥\varsigma ∥ \end{array}$$

where $\zeta_{4}=N∥B_{L}∥{∥\Delta ∥}_{\infty }+\bar{\varphi }\left(B_{L}\right)\underline{\varphi }\left(B_{L}\right){∥G\left(x\right)∥}_{F}∥\lambda_{0}∥{∥\Delta ∥}_{\infty }∥V_{0}\left(t_{k}\right)∥$. In the next term, $\varsigma_{i}\left(t\right)-\varsigma_{i}\left(t_{k}\right)$ is obtained so that

(A16) $$\begin{array}{cc} \; & \varsigma_{i}\left(t\right)-\varsigma_{i}\left(t_{k}\right)\\ = & c_{i}\cdot e_{ix}\left(t\right)+e_{iv}\left(t\right)-\varrho_{i}\left(t\right)-c_{i}\cdot e_{ix}\left(t_{k}\right)-e_{i\nu }\left(t_{k}\right)+\varrho_{i}\left(t_{k}\right)\\ \leq & ∥B_{L}∥\left(c_{i}\cdot \left(∥\Delta_{ix}∥+∥\Delta_{0x}∥\right)+\left(∥\Delta_{i\nu }∥+∥\Delta_{0\nu }∥\right)\right)\\ \leq & ∥B_{L}∥\left(c_{i}+1\right)\left({∥\Delta_{i}∥}_{\infty }+{∥\Delta_{0}∥}_{\infty }\right)=\vartheta_{i} \end{array}$$

where $\vartheta_{i}=∥B_{L}∥\left(c_{i}+1\right)\left({∥\Delta_{i}∥}_{\infty }+{∥\Delta_{0}∥}_{\infty }\right)$. According to the bound of $\varsigma_{i}\left(t\right)-\varsigma_{i}\left(t_{k}\right)$ and $\varsigma_{i}\left(t\right)$, one can obtain that

(A17) $$\begin{array}{cc} \; & \varsigma_{i}^{2}\left(t\right)-\varsigma_{i}^{2}\left(t_{k}\right)\\ = & \left(\varsigma_{i}\left(t\right)-\varsigma_{i}\left(t_{k}\right)\right)\left(\varsigma_{i}\left(t\right)+\varsigma_{i}\left(t_{k}\right)\right)\\ \leq & 2\vartheta_{i}{∥\varsigma \left(t\right)∥}_{\infty } \end{array}$$

Then, ${\hat{\phi }}_{i}-{\hat{\phi }}_{i}\left(t_{k}^{i}\right)$ is obtained by integrating the adaptive adaptive minimum parameter learning mechanism (17) from $t_{k}^{i}$ to *t*, where $t\in \left[t_{k}^{i},t_{k+1}^{i}\right)$, so that

(A18) $$\begin{array}{cc} \; & {\hat{\phi }}_{i}-{\hat{\phi }}_{i}\left(t_{k}^{i}\right)\\ = & -∥B_{L}∥H_{i}\left(x\right)\int_{t_{k}^{i}}^{t}\frac{\gamma }{2}\varsigma_{i}^{2}\left(t\right)dt+ky\int_{t_{k}^{i}}^{t}{\hat{\phi }}_{i}\left(t\right)dt\\ \leq & 2∥B_{L}∥\left(H_{i}\left(x_{k}^{i}\right)+\lambda_{3i}∥\Delta_{x}∥\right)\vartheta ∥\varsigma \left(t\right)∥T-kyT\left({\hat{\phi }}_{i}-{\hat{\phi }}_{i}\left(t_{k}^{i}\right)\right)\\ \Rightarrow & {\hat{\phi }}_{i}-{\hat{\phi }}_{i}\left(t_{k}^{i}\right)\leq \frac{2}{1+kyT}\delta_{1}^{i}\vartheta_{i}∥B_{L}∥∥\varsigma \left(t\right)∥T \end{array}$$

where $\delta_{1}^{i}=H_{i}\left(x_{k}^{i}\right)+\lambda_{3i}∥\Delta_{i}∥$. Denoting ${\bar{g}}_{i}\left(t\right)={\hat{\phi }}_{i}H_{i}\varsigma_{i}$, the following inequality is written as

(A19) $$\begin{array}{cc} \; & {\bar{g}}_{i}\left(t\right)-{\bar{g}}_{i}\left(t_{k}\right)=H_{i}\int_{t_{k}^{i}}^{t}\dot{\hat{\phi }}\left(t\right)\varsigma_{i}\left(t\right)dt\\ \leq & \delta_{1}^{i}\left(\hat{\phi }\left(t_{k}^{i}\right)+\frac{1}{1+kyT}2\delta_{1}^{i}\vartheta_{i}∥B_{L}∥∥\varsigma \left(t\right)∥T\right)\vartheta_{i}T \end{array}$$

Let $\delta_{3}^{i}=\delta_{1}^{i}\left(\hat{\phi }\left(t_{k}^{i}\right)+2\delta_{1}^{i}\vartheta_{i}∥B_{L}∥∥\varsigma \left(t\right)∥T/\left(1+k\gamma T\right)\right)\vartheta_{i}T$

(A20) $$\begin{array}{cc} \; & {\hat{g}}_{i}\left(x\right){\bar{g}}_{i}\left(t\right)-{\hat{g}}_{i}\left(x_{k}^{i}\right){\bar{g}}_{i}\left(t_{k}^{i}\right)\\ = & {\hat{g}}_{i}\left(x\right){\bar{g}}_{i}\left(t\right)-{\hat{g}}_{i}\left(x\right){\bar{g}}_{i}\left(t_{k}^{i}\right)+{\hat{g}}_{i}\left(x\right){\bar{g}}_{i}\left(t_{k}^{i}\right)-{\hat{g}}_{i}\left(x_{k}^{i}\right){\bar{g}}_{i}\left(t_{k}^{i}\right)\\ \leq & \delta_{3}^{i}\left({\hat{g}}_{i}\left(x\right)+\lambda_{i0}{∥\Delta_{i}∥}_{\infty }\right)+{\bar{g}}_{i}\left(t_{k}^{i}\right)\lambda_{i0}{∥\Delta_{i}∥}_{\infty } \end{array}$$

Thus, the next term of ${\dot{L}}_{4}$ is rewritten as

(A21) $$\begin{array}{cc} \; & \frac{1}{2}\varsigma^{T}B_{L}\hat{\phi }H\varsigma -\frac{1}{2}\varsigma^{T}B_{L}G\left(x\right)G^{-1}\left(x_{k}\right)B_{L}^{-1}\hat{\phi }\left(t_{k}\right)H\left(x_{k}\right)\varsigma \left(t_{k}\right)\\ \leq & \frac{1}{2}∥B_{L}∥∥\delta_{3}∥∥\varsigma ∥+\frac{1}{2}∥B_{L}∥∥G\left(x\right)∥{}_{F}∥\lambda_{0}{∥∥\Delta ∥}_{\infty }{∥H\left(x_{k}\right)∥}_{F}∥\varsigma \left(t_{k}\right)∥∥\varsigma ∥\\ = & \zeta_{5}∥\varsigma ∥ \end{array}$$

where $\zeta_{5}=\frac{1}{2}∥B_{L}∥∥\delta_{3}∥+\frac{1}{2}∥B_{L}∥∥G\left(x\right)∥{}_{F}∥\lambda_{0}{∥∥\Delta ∥}_{\infty }{∥H\left(x_{k}\right)∥}_{F}∥\varsigma \left(t_{k}\right)∥$. Applying the transformation of the last two terms of ${\dot{L}}_{3}$, (A15) is obtained as

(A22) $$\begin{array}{cc} \; & -\varsigma^{T}B_{L}G\left(x\right)G^{-1}\left(x_{k}\right)B_{L}^{-1}\mathrm{sgn}\left(\varsigma \left(t_{k}\right)\right)\\ = & \varsigma^{T}B_{L}G\left(x\right)G^{-1}\left(x_{k}\right)B_{L}^{-1}\left(\mathrm{sgn}\left(\varsigma \left(t\right)\right)-\mathrm{sgn}\left(\varsigma \left(t_{k}\right)\right)\right)\\ \; & -\varsigma^{T}B_{L}G\left(x\right)G^{-1}\left(x\right)B_{L}^{-1}\left(\mathrm{sgn}\left(\varsigma \left(t\right)\right)-\mathrm{sgn}\left(\varsigma \left(t_{k}\right)\right)\right)\\ \; & +\varsigma^{T}\left(\mathrm{sgn}\left(\varsigma \left(t\right)\right)-\mathrm{sgn}\left(\varsigma \left(t_{k}\right)\right)\right)+\varsigma^{T}B_{L}G\left(x\right)G^{-1}\left(x\right)B_{L}^{-1}\mathrm{sgn}\left(\varsigma \left(t\right)\right)\\ \; & -\varsigma^{T}B_{L}G\left(x\right)G^{-1}\left(x_{k}\right)B_{L}^{-1}\mathrm{sgn}\left(\varsigma \left(t\right)\right)-\varsigma^{T}\mathrm{sgn}\left(\varsigma \left(t\right)\right)\\ \leq & 3\bar{\varphi }\left(B_{L}\right)/\varphi \left(B_{L}\right){∥G\left(x\right)∥}_{F}∥\lambda_{0}∥{∥\Delta ∥}_{\infty }∥\varsigma ∥-∥\varsigma ∥\\ = & \zeta_{6}∥\varsigma ∥-∥\varsigma ∥ \end{array}$$

where $\zeta_{6}=3\bar{\varphi }\left(B_{L}\right)/\varphi \left(B_{L}\right){∥G\left(x\right)∥}_{F}∥\lambda_{0}∥{∥\Delta ∥}_{\infty }$. In a similar way, (A16) is obtained as

(A23) $$\begin{array}{cc} \; & -\varsigma^{T}B_{L}G\left(x\right)G^{-1}\left(x_{k}\right)B_{L}^{-1}\varsigma \left(t_{k}\right)\\ \leq & \bar{\varphi }\left(B_{L}\right)/\underline{\varphi }\left(B_{L}\right)∥\theta ∥{∥G(x)∥}_{F}∥\lambda_{0}∥{∥\Delta ∥}_{\infty }∥\varsigma ∥\\ \; & +\bar{\varphi }\left(B_{L}\right)/\underline{\varphi }\left(B_{L}\right){∥G(x)∥}_{F}∥\lambda_{0}∥{∥\Delta ∥}_{\infty }{∥\varsigma ∥}^{2}-{∥\varsigma ∥}^{2}\\ = & \left(\zeta_{7}+\zeta_{8}\right)∥\varsigma ∥-{∥\varsigma ∥}^{2} \end{array}$$

where $\zeta_{7}=\bar{\varphi }\left(B_{L}\right)/\underline{\varphi }\left(B_{L}\right){∥\theta ∥∥G\left(x\right)∥}_{F}∥∥\lambda_{0}∥{∥\Delta ∥}_{\infty }$ and $\zeta_{8}=\bar{\varphi }\left(B_{L}\right)/\underline{\varphi }\left(B_{L}\right){∥G(x)∥}_{F}∥\lambda_{0}∥{∥\Delta ∥}_{\infty }∥\varsigma ∥$. Relying on (A7), (A13)–(A15) and (A21)–(A24), the Lyapunov function is derived as

(A24) $$\begin{array}{cc} \; & {\dot{L}}_{3}={\dot{L}}_{4}+{\dot{L}}_{5}\\ \leq & ∥B_{L}∥\left(\left(∥\sigma ∥∥\varsigma ∥+\frac{1}{2}N\right)+\frac{1}{2}\mathrm{tr}\left\{{\tilde{\phi }}^{T}\varsigma \varsigma^{T}H\right\}\right)-∥\eta ∥∥\varsigma ∥\\ \; & -{∥\mu ∥∥\varsigma ∥}^{2}+\left(\zeta_{1}+\zeta_{2}+\zeta_{3}+\zeta_{4}+\zeta_{5}\right.\\ \; & \left.+\zeta_{6}+\zeta_{7}+\zeta_{8}\right)∥\varsigma ∥+\frac{1}{\gamma }\mathrm{tr}\left\{\tilde{\phi }\dot{\phi }\right\} \end{array}$$

Using a positive value $∥\eta ∥$ ensures that

(A25) $$∥\eta ∥=\left(\zeta_{1}+\zeta_{2}+\zeta_{3}+\zeta_{4}+\zeta_{5}+\zeta_{6}+\zeta_{7}+\zeta_{8}+\tau \right)$$

(A26) $${\dot{L}}_{3}\leq -∥\mu ∥{∥\varsigma ∥}^{2}-\kappa {∥\hat{\phi }∥}_{F}{∥\tilde{\phi }∥}_{F}+\frac{N}{2}∥B_{L}∥$$

Choosing $\kappa =\frac{2∥\mu ∥}{\gamma }$, we have

(A27) $${\dot{L}}_{3}\leq -2∥\mu ∥L_{3}+Q_{2}$$

where $Q_{2}=\frac{N}{2}∥B_{L}∥+\frac{\kappa }{2}{∥\phi ∥}_{F}^{2}$. According to Lemma 1.1 in Ge and Wang (2004), $L\left(t\right)$ is bounded and the differential inequality function is solved as

(A28) $$L_{3}\leq \frac{Q}{2R}+\left(L_{3}\left(0\right)-\frac{Q}{2R}\right)e^{-2Rt}$$

(A29) $$\lim_{t\to \infty }L_{3}=\frac{Q}{2R}=\frac{\frac{\kappa }{2}{∥\phi ∥}_{F}^{2}+\frac{1}{2}N∥B_{L}∥}{2R}=\frac{{∥\phi ∥}_{F}^{2}}{2\gamma }+\frac{N∥B_{L}∥}{4R}$$

Similarly, when the time reaches infinity, the Lyapunov function approaches a bounded value and asymptotic stability is achieved from the inequality (A29). When the state trajectories approach the neighbor of the sliding surface, $sign\left(\varsigma_{i}\left(t\right)\right)\neq sign\left(\varsigma_{i}\left(t_{k}^{i}\right)\right)$ is observed. We move to the second case. It is obtained from the definitions of the measurement errors (22) and (23) that

(A30) $$\begin{array}{cc} \varsigma (t)= & \omega \varepsilon_{1}\left(t\right)+\varepsilon_{2}\left(t\right)-\varrho \left(t\right)\\ = & \omega B_{L}\left[\tilde{x}\left(t_{k}\right)-\Delta_{x}+\Delta_{0x}⊗I_{N}\right]\\ \; & +B_{L}\left[\tilde{v}\left(t_{k}\right)-\Delta_{v}+\Delta_{0v}⊗I_{N}\right]-\varrho \left(t\right)+\varrho \left(t_{k}\right)\\ = & \varsigma \left(t_{k}\right)-\omega B_{L}\left(\Delta_{x}-\Delta_{0x}⊗I_{N}+\Delta_{v}-\Delta_{0v}⊗I_{N}\right)-\left(\varrho \left(t\right)-\varrho \left(t_{k}\right)\right) \end{array}$$

Then, the bound of the sliding mode function is deduced as

(A31) $$\begin{array}{cc} \left|\varsigma_{i}\left(t\right)\right|\leq & ∥B_{L}∥\left(∥\omega ∥\left(∥\Delta_{ix}∥+∥\Delta_{0x}∥\right)+\left(∥\Delta_{i\nu }∥+∥\Delta_{0\nu }∥\right)\right)\\ \leq & ∥B_{L}∥\left(∥\omega ∥+1\right)\left({∥\Delta_{i}∥}_{\infty }+{∥\Delta_{0}∥}_{\infty }\right)=\vartheta \end{array}$$

According to the above analysis, regardless of whether the agent states are beyond the bound $sign\left(\varsigma_{i}\left(t\right)\right)\neq sign\left(\varsigma_{i}\left(t_{k}^{i}\right)\right)$, the states of the agents are constrained to the bound. □

## Appendix C. Proof of Theorem 3

**Proof.** A positive constant $\lambda_{4i}$ that satisfies $\varsigma_{i}^{2}\left(t\right)-\varsigma_{i}^{2}\left(t_{k}^{i}\right)\leq \lambda_{4i}\left|t-t_{k}^{i}\right|=\lambda_{4i}T_{k}^{i}$ exists. In addition, a positive constant $\lambda_{5i}$ that satisfies ${\bar{w}}_{i}-{\bar{w}}_{i}\left(x_{k}^{i}\right)=\lambda_{5i}{∥x-x_{k}^{i}∥}_{\infty }$ exists.

(A32) $$\begin{array}{cc} \frac{d}{dt}∥\Delta_{i}\left(t\right)∥ & =∥{\dot{\Delta }}_{i}\left(t\right)∥\\ \; & ={∥\left[\begin{array}{c} v_{i}\left(t_{k}^{i}\right)-\Delta_{iv}\left(t\right)\\ f_{i}\left(x_{i}\right)+g\left(x_{i}\right)u_{i}\left(t_{k}^{i}\right)+d_{i}\left(t\right) \end{array}\right]∥}_{\infty } \end{array}$$

(A33) $$\begin{array}{cc} \; & \frac{d}{dt}∥\Delta_{i}\left(t\right)∥\\ \leq & ∥v_{i}\left(t_{k}^{i}\right)∥+∥\Delta_{i}\left(t\right)∥+∥g\left(x_{i}\right)∥∥g^{-1}\left(x_{i}\right)f_{i}\left(x_{i}\right)\\ \; & +\sum_{i=1}^{N}u_{j}\left(t_{k}^{i}\right)+g^{-1}\left(x_{i}\right)d_{i}\left(t\right)\\ \; & +g_{i}^{-1}\left(x\left(t_{k}^{i}\right)\right)\left(-\frac{1}{2}s_{i}\left(t_{k}^{i}\right)\hat{\phi }\left(t_{k}^{i}\right)h_{i}^{T}h_{i}+v_{0}\left(t_{k}^{i}\right)\right.\\ \; & -c_{i}e_{ix}\left(t_{k}^{i}\right)-f_{i}\left(x\left(t_{k}^{i}\right)\right)-\mu_{i}\varsigma_{i}\left(t_{k}^{i}\right)+\varrho_{i}\left(t_{k}^{i}\right)∥ \end{array}$$

(A34) $${\bar{\lambda }}_{0}=1+∥g_{i}\left(x\right)∥∥\lambda_{5}∥$$

Relying on (A10)–(A12), the following inequalities are given as

(A35) $$\begin{array}{cc} \hat{\phi }-\hat{\phi }\left(t_{k}^{i}\right)= & \int_{t_{k}}^{t}\left(\frac{\gamma }{2}h_{i}^{T}h_{i}\varsigma_{i}^{2}-\kappa \gamma \phi \right)dt\\ \leq & \frac{\gamma T_{k}^{i}}{2\left(1+\kappa \gamma T_{k}^{i}\right)}\left(h_{i}^{T}\left(x_{ik}\right)h_{i}\left(x_{ik}\right)\right.\\ \; & \left.+\lambda_{3i}∥\Delta_{ik}∥\right)\left(\varsigma_{i}^{2}\left(t_{k}^{i}\right)+\lambda_{4i}T_{k}^{i}\right)=\delta_{4} \end{array}$$

(A36) $$\begin{array}{cc} \bar{m}-\bar{m}\left(t_{k}^{i}\right)= & \frac{1}{2}h_{i}^{T}h_{i}\int_{t_{k}}^{t}{\hat{\phi }}_{i}\varsigma_{i}dt\\ \leq & \frac{1}{2}\left(\lambda_{3i}{∥\Delta_{ix}∥}_{\infty }+h_{i}^{T}\left(x_{ik}\right)h_{i}\left(x_{ik}\right)\right)\\ \; & \left({\hat{\phi }}_{i}\left(t_{k}^{i}\right)+\delta_{4}\right)\left(\varsigma_{i}\left(t_{k}^{i}\right)+\vartheta_{i}\right)T_{k}^{i}=\delta_{5} \end{array}$$

Let $\Lambda \left(t\right)=v_{0}\left(t\right)-c_{i}e_{ix}\left(t\right)-\mu_{i}\varsigma_{i}\left(t\right)$, and then

(A37) $$\begin{array}{cc} \frac{d}{dt}∥\Delta_{i}\left(t\right)∥\leq & ∥v_{i}\left(t_{k}^{i}\right)∥+∥\Delta_{i}\left(t\right)∥+∥g\left(x_{i}\right)∥\\ \; & \left(∥L_{6i}∥∥\Delta_{i}\left(t\right)∥+\delta_{5}+∥\sum_{i=1}^{N}u_{j}\left(t_{k}^{i}\right)∥+\frac{1}{2}\right.\\ \; & \left.+∥g_{i}^{-1}\left(x\left(t_{k}^{i}\right)\right)∥\left(∥\Lambda \left(t_{k}^{i}\right)∥+\varsigma_{i}\left(0\right)\right)\right) \end{array}$$

For convenience, $\bar{n}$ is defined as

(A38) $$\begin{array}{cc} \bar{n}= & ∥v_{i}\left(t_{k}^{i}\right)∥+∥g\left(x_{i}\right)∥\delta_{5}+∥g\left(x_{i}\right)∥\left(∥\sum_{i=1}^{N}u_{j}\left(t_{k}^{i}\right)∥\right.\\ \; & \left.+\frac{1}{2}+∥g_{i}^{-1}\left(x\left(t_{k}^{i}\right)\right)∥\left(∥\Lambda \left(t_{k}^{i}\right)∥+\varsigma_{i}\left(0\right)\right)\right) \end{array}$$

The differential inequality equation is solved as

(A39) $$∥\Delta_{i}\left(t\right)∥\leq \bar{n}/{\bar{\lambda }}_{0}\left(e^{{\bar{\lambda }}_{0}t-t_{k}^{i}}-1\right)$$

It is concluded that a positive bound exists between two time intervals and the Zeno behavior is excluded. □
